# A cardinal approach to evaluating the effectiveness of psychosocial rehabilitation based on the analytical hierarchy process

**DOI:** 10.1192/j.eurpsy.2022.1944

**Published:** 2022-09-01

**Authors:** V. Mitikhin, T. Solokhina, G. Tiumenkova, M. Kuzminova

**Affiliations:** FSBSI “Mental Health Research Centre”, Department Of Mental Health Services, Moscow, Russian Federation

**Keywords:** rehabilitation, analytic hierarchy process, effectiveness, psychosocial

## Abstract

**Introduction:**

The approach to evaluating the effectiveness of psychosocial rehabilitation (PSR) of mentally ill patients implies a multidimensional, hierarchical consideration of mental pathology with the inclusion of clinical and psychopathological, socio-environmental and personal indicators when setting goals and objectives of PSR

**Objectives:**

Development of algorithms and models for evaluating the effectiveness of PSR taking into account: clinical, socio-demographic, psychological characteristics of the patient and the characteristics of the family environment, as well as factors of psychiatric care.

**Methods:**

Clinical, statistical, algorithms of the analytical hierarchy process (AHP) [1].

**Results:**

Numerical estimates of changes (before and after the PSR program) in the main areas of patient’s functioning disorders, such as motivation, cognition, compliance, coping strategies, family, skills, immediate environment, and others, are proposed as particular criteria for evaluating the effectiveness of PSR. Quantitative estimates for particular criteria are obtained on the basis of rank estimates, which are converted into numerical ones based on AHP algorithms [1]. Quantitative integral estimates of the effectiveness of PSR are obtained on the basis of partial estimates, taking into account the weight of the corresponding areas of impaired functioning of patients.

**Conclusions:**

The developed approach opens up prospects for obtaining numerical, partial and integral estimates based on various rank scales, which are of interest from the point of view of forming criteria-indicators (markers) of the effectiveness of psychosocial, rehabilitation, psychoeducational and psychotherapeutic measures. References: 1. Mitikhin, V.G., Solokhina, T.A. *S.S. Korsakov Journal of Neurology and Psychiatry.* 2019; 119(2): 49-54

**Disclosure:**

No significant relationships.

